# Experimental and computational studies of novel cyclic ammonium based ionic liquids as corrosion inhibitors for carbon steel in acid medium

**DOI:** 10.1038/s41598-024-61368-w

**Published:** 2024-05-20

**Authors:** Raghda A. El-Nagar, Maher I. Nessim, N. A. Khalil, Safaa I. Elewa

**Affiliations:** 1 Petroleum Testing Lab, Analysis and Evaluation Department, Egyptian Petroleum Research Institute, Nasr City, Cairo 11727 Egypt; 2 Department of Chemistry, Faculty of Women, Ain Shams University, Heliopolis, Cairo Egypt

**Keywords:** Cyclic ammonium ionic liquids, Corrosion, Acidic media, Efficiency, DFT, Thermodynamic, Chemistry, Electrochemistry

## Abstract

The challenge of corrosion posed as a result of acidic sittings is considered as a major industrial concern, wherein ionic liquids serve as crucial in addressing the corrosive impacts on metals. In this study, five selected cyclic ammonium based ionic liquids were synthesized; IL-1MPyrBr, IL-1MPipBr, IL-2PyBr, IL-3MPyBr and IL-4MPyBr and their chemical structures were characterized using a variety of spectroscopic techniques (FT-IR, ^I^H-NMR, ^13^C-NMR, Elemental analysis and thermal gravimetric analysis (TGA). Their corrosion inhibition efficiency was studied on carbon steel in 1 M HCl via different concentrations at 298 K using chemical and electrochemical parameters (PDP and EIS). DFT quantum parameters were computed, and the noted results were in complete compatible with the experimental. The synthesized ILs recorded excellent inhibition on the carbon steel corrosion in acidic media with increasing efficiency by increasing the inhibitor concentrations from 20 to 100 ppm. Different cations in the synthesized ILs affect the anti-corrosion effect and IL-3MPyBr showed the highest inhibition (ηR); 96.12% using the lowest concentration. Kinetic and thermodynamic considerations were studied and illustrated.

## Introduction

Carbon steels are frequently used in the petroleum industry especially petroleum products related issues; such as for their financial benefits and exceptional physical features, in oil products including pipeline systems, petroleum exploration facilities, oil refining processes^1–5^. Particularly, through the crude oil transportation, pipelines may experience interior corrosion, which reduces their service life and causes significant resource waste and financial losses^6–8^. The lengthy life of the pipeline is severely impacted by the corrosive character of the fluids it carries, complicated flow conditions, and sporadic solid particle presence. This causes early localized perforation and corrosion. For instance, pitting may occur and the passivation film on the surface of mild steel may dissolve when certain aggressive anions, like chloride, migrate into the active corrosion zone^9,11^. Internal corrosion is mostly prevented in a variety of industries, most notably the oil and gas sector, by corrosion inhibitors because of acid treatment and acidic, moist environments. Corrosion inhibitors are added either constantly or semi-continuously, with a concentration of around one part per million, without stopping the process.

According to the literature^11^, cathodic protection, surface manipulation of metal, and synthetic corrosion inhibitors can all be used to prevent corrosion in carbon steel. Synthetic corrosion inhibitors, which are one of these because of their affordability and simplicity of application, are among the most efficient ways to lessen corrosion in industrial operations^12^. Commonly employed as corrosion inhibitors and potentially damaging to the environment are chemical chemicals, primarily organic ones including tungstates, molbdates, vanadates and chromates. As a result, demand for eco-friendly chemicals is growing^13–15^.

At the water-metal contact, water is replaced by hydrophobic tails, which also repel the electrolyte, and the polar group of the corrosion inhibitor, which has a great affinity for both. The use of organic compounds as corrosion inhibitors for metal surfaces has been well documented^16–20^ because they can combine with heteroatoms such O, N, P, and S-bonds. Non-toxic corrosion inhibitors are desperately needed because organic traditional inhibitors have a negative impact on the surrounding environment. Biopolymers, surfactants, amino acids, natural products, lanthanides and ionic liquids (ILs)^21–25^, all are proposed as novel environmentally benign corrosion inhibitors. ILs are made up of ions, normally melts at ambient temperatures (below 100 °C). They are mostly non-flammable, have approximately negligible vapor pressure, and has exceptional thermal stability when compared to organic solvents^26–28^ ILs possess no vapor-related hazards because they are often harder to break down and vaporize than other potential green corrosion inhibitors.

The world corrosion prevention is constantly growing, with new discoveries happening all the time. Scientists are particularly excited about organic heterocyclic molecules with special atoms like sulfur, nitrogen, and oxygen in their structure. These molecules work their magic at the meeting point between metal and solution, where they form a protective layer. This layer stops the metal from deteriorating by clinging to the surface. The secret to their success lies in how they interact with the metal using special donor atoms, electron pathways, and the overall structure of the molecule itself. In acidic environments, these heterocyclic molecules can even take on a positive charge, making them even better at stopping metal from corrosion^29–31^.”

In 2017, Sugirtha Velusamy et al.^32^ investigated the potential of imidazolium based ILs as corrosion inhibitors for mild steel in variety of fluids; CaCl2, HCOOCs and ZnBr_2_. Resulting from different methodologies ILs suggested to explore and develop anti-corrosive completion fluids suitable for oil and gas reservoir areas. Ardakani et al. in 2021^26^, summarized the corrosion inhibition properties of various ionic liquid inhibitor compounds based on different cations into different corrosive environments acidic, basic, salty and aqueous media. According to Ardakani's analysis, numerous ionic liquid particularly which based on imidazolium cations notable effectiveness as corrosion inhibitors attributing to presence of heteroatoms with free pair electrons (O, S, N, P, etc.), pi-electron, polar functional groups and the conjugated system. Also in 2021, Kobzar et al.^31^ aimed is to assess the advanced trends in developing the different types of ILs in the corrosion inhibition field over the past three years. Kobzar`s review based on molecular mass, ionic nature and chemical structure of ILs. Haldhar et al. in 2023^13^, noted that the corrosion inhibitors with extending alkyl chains of enhance the inhibition performance, all these expectations were confirmed using experimental and theoretical calculations. Jia et al. 2024^33^, synthesized phosphorus-based ionic liquids (PBILs) with different alkyl chain length and evaluated their anticorrosion efficiencies for mild steel (MS) in 1 M HCl aqueous solution physically and theoretically. The results revealed that PBILs performed excellently (inhibition efficiencies > 93%).

These data led some researchers to suggest that ILs are the best substance for preventing corrosion in metals^34,35^. Investigations into ILs corrosion inhibitors are growing fast from a different perspective. In our research, to contribute to this hot research domain, this work focus on the creation, confirmation, and application of a newly designed series of cyclic ammonium-based ionic liquids, assessed as inhibitors for carbon steel corrosion in 1M HCl solution. This evaluation employs Electrochemical Impedance Spectroscopy (EIS) and Potentiodynamic Polarization (PDP) methods at various inhibitor concentrations ranging from 20 to 100 ppm, alongside Density Functional Theory (DFT) computational calculations. Furthermore, we explore and discuss both kinetic and thermodynamic parameters. Additionally, as a semi-pilot bench scale for potential industrial applications, cleaning processes are executed to evaluate the anti-corrosive efficacy of the synthesized ionic liquids.

## Experimental

### Materials

The aforementioned chemicals are being used to synthesize the targeted ILs without further purifications: Naphthalen-2-ol (99%), potassium hydroxide (97%), Ethanol (99%), dibromoethan (98%), 1-methylpyrrolidine (97%), 1-methylpiperidine (99%), pyridine (98%), 3-methylpyridine (98%), 4-methylpyridine (99%) and acetonitrile (99%). All the used chemicals were delivered from Alfa Aesar and Sigma Aldrich companies.

### Synthesis of Ionic liquids

#### Preparation of 2-(2-bromoethoxy) naphthalene (I)

**Figure 1:**
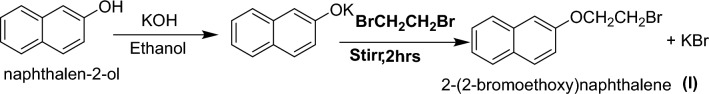
Preparation of 2-(2-bromoethoxy) naphthalene.

Equivalent amount of Naphthalen-2-ol and potassium hydroxide 0.1mol were dissolved in 50 mL of Ethanol with stirring. Over around two hours and at 25 °C, 0.01 mol of dibromoethane was gradual added; drop by drop to a stirring solution and replaced in the manuscript as clear using track changes. KBr precipitate (byproduct) was elevated and the white solid product was obtained by removing the solvent under vacuum. The produce was washed, using deionized water and then recrystallized from ethanol. Figure 1 The melting-point was recorded with complete compatible comparing with those in literatures; 80–82 °C^35^.

#### Preparation of IL-1MPyrBr, IL-1MPipBr, IL-2PyBr, IL-3MPyBr, IL-4MPyBr

**Figure 2:**
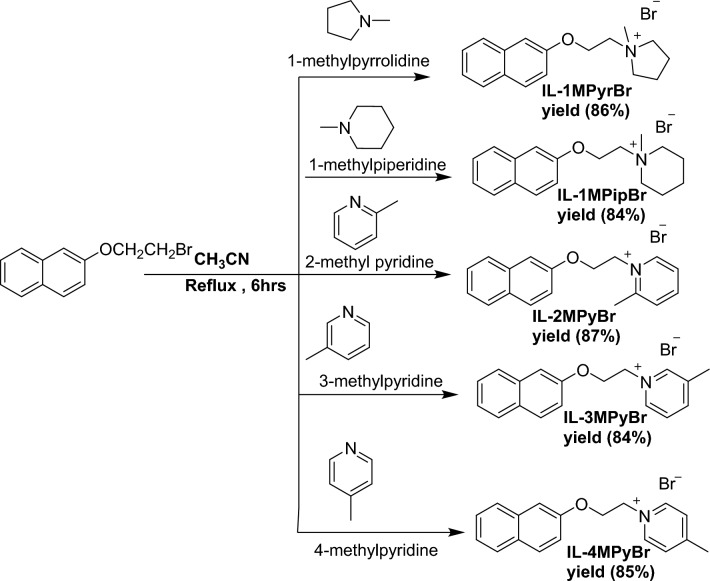
Preparation of IL-1MPyrBr, IL-1MPipBr, IL-2PyBr, IL-3MPyBr, IL-4MPyBr.

Different cyclic amines (1-methylpyrrolidine, 1-methylpiperidine, 2-methyl pyridine, 3-methylpyridine, and 4-methylpyridine) were refluxed with solution of 2-(2-bromoethoxy) naphthalene (I) in presence of 50 ml CH_3_CN for six hours at 80°C. The products were concentrated and evaporated under vacuum Fig. 2.

The chemical structure confirmation for the synthesized ILs was carried out through different techniques. Elemental analysis (Perkin Elmer 2400 CHN elemental analyzer. Waltham, MA). FT-IR (Perkin-Elmer-1430 infrared spectrophotometer, Waltham, MA) by using the potassium bromide wafer method. ^1^H-NMR and ^13^C spectra were determined via a burker advance III 400 MHz (high performance digital FT-NMR spectrometer) using dimethyl sulfoxide (DMSO)-d6 as a solvent. Chemical shifts were noted in d (ppm) regarding to tetramethylsilane (TMS) as an internal standard structures (TMS). Thermal gravimetric analysis was determined via the thermal analyzer at heating rate of 10 °C/min. samples were heated from room temperature to 600 °C under flow of nitrogen.

### Evaluation of the inhibitory performance

#### Electrochemical methods

In the context of electrochemical assessments, a glass cell with a capacity of 100 ml was employed, equipped with three electrodes. The experimental setup comprised a platinum sheet serving as the counter electrode, and a saturated calomel electrode (SCE) as the reference electrode. As the working electrode, carbon steel with a surface area of 0.345 cm^2^ was utilized. All experimental procedures were conducted using an OrigaMaster 5potentiostat/galvanostat. Potential current plots were generated under specific conditions, including a scan rate of 1.0 mV s^−1^ and a potential range of ± 250 mV versus OCP (Open Circuit Potential) Furthermore, electrochemical impedance spectroscopy (EIS) tests were conducted at OCP within specific parameters, involving a frequency range of 100 kHz to 0.1 Hz and an AC voltage amplitude of 20 mV. The construction of the equivalent circuit was facilitated by the utilization of the OrigaMaster 5 software. Density Function Theory (DFT) for the MNDO semi empirical process was used to determine the quantum parameters for the synthesized ILs theory research. All the computational studies were carried out using GAUSSIAN 09 revision-D.01-SMP (DFT).

#### Weight loss method

Carbon steel sheets, employed for corrosion testing, have a consistent exposed surface area of 0.345 cm^2^. The sheets underwent abrasion using a series of emery papers and were subsequently cleaned following the G1-03/ASTM standard method. Prior to immersion in 100 ml of an HCl solution at preset concentrations for 180 min within temperature ranges of 298, 313 and 323 K, the specimens were accurately weighed. To examine the impact on corrosion rates, varying doses of IL-3MPyBr, the selected ionic liquid for the study, were introduced into the acidic solutions. The prepared solutions were then exposed to the atmosphere, removed at specific intervals, cleaned, dried, and meticulously reweighed. Each experiment was conducted in triplicate, involving the weighing of three carbon steel sheets, and the resulting averages were calculated.

#### Theoretical studies

For the purpose of studying the various inhibitory efficiencies as well as the reactive sites of the synthesized ILs as corrosion inhibitors, a theoretical analysis of IL-1MPyrBr, IL-1MPipBr, IL-2PyBr, IL-3MPyBr, and IL-4MPyBr was carried out. The determination of corrosion inhibitors was in relation to several parameters such as dipole moment (λ), highest occupied molecular orbital energy (E_HOMO_), lowest unoccupied molecular orbital energy (E_LUMO_) and energy gap (ΔE). All parameters were calculated regarding to DFT theory using the GAUSSIAN 09 Revision-D.01-SMP programs^36–38^.

## Results and discussion

### Confirmation of the synthesized ILs

#### 1-methyl-1-(2-(naphthalen-2-yloxy) ethyl) pyrrolidin-1-ium Bromide (IL-1MPyrBr)

IR (KBr, ʋ cm^−1^): 1214 (C–O), 1389–1457 (C–N), 1598 (C=C), 2943 (CH Aliphatic), 3050 (CH Aromatic);^1^ H NMR (DMSO-*d*_6_) δ ppm: naphthalene protons 7.22–7.38 (m, 7H), spacer protons 4.5 (t, 2H, O–CH_2_), 3.6 (t, 2H, O–CH_2_ CH_2_), 3.9 (s,3H,CH_3_), pyrrolidinium protons 2.06 (t, 4H), 3.16 (t, 4H); ^13^C-NMR (DMSO-*d*_6_) δ ppm: 156.09 (C=C–O), naphthalene (134.93–107.83), spacer 64.86 (O–CH_2_), 62.77 (O–CH_2_–CH_2_), 48.58 (CH_3_) pyrrolidinium carbons (21.08, 21.41, 62.17); Anal. Calcd for C_17_H_22_BrNO + (335.09): C, 60.72; H, 6.59; Br, 23.76; N, 4.17; O, 4.76; Found: 60.03; H, 6.49; N, 4.21.

#### 1-methyl-1-(2-(naphthalen-2-yloxy) ethyl) piperidin-1-ium Bromide (IL-1MPipBr)

IR (KBr, ʋ cm^−1^): 1214 (C–O), 1389–1457 (C–N), 1598 (C=C), 2943 (CH Aliphatic), 3050 (CH Aromatic); ^1^H NMR (DMSO-d^6^) δ ppm: naphthalene protons 7.18–7.89 (m, 7H), spacer protons 4.6 (t, 2H, O CH_2_), 4.46 (t, 2H, CH_2_ CH_2_), piperidinium protons 1.57 (t, 2H), 1.87 (t, 4H), 3.19 (t, 4H), 3.8 (s, 3H); ^13^C-NMR (DMSO-*d*_6_) δ ppm: 155.76 (C=C–O), naphthalene (134.16–107.14), spacer 67.78 (O–CH_2_), 61.34 (O–CH_2_–CH_2_), 49.99 (CH_3_) piperidinium carbons (20.55, 19.36, 60.89) ; Anal. Calcd for C18H24BrNO + (349.10): C, 61.72; H, 6. 91; Br, 22.81; N, 4.00; O, 4.57; Found: 61.51; H, 6.70; N, 4.45.

#### 2-methyl-1-(2-(naphthalen-2-yloxy) ethyl) pyridin-1-ium bromide (IL-2MPyBr)

IR (KBr, ʋ cm^−1^ 1213 (C–O), 1389–1455 (C–N), 1598 (C=C), 2950 (CH Aliphatic), 3066 (CH Aromatic);^1^ H NMR (DMSO-d6) δ ppm: pyridinium protons 7.84–9.76 (m, 4H), naphthalene protons 7.17–7.67 (m, 7H), spacer protons 5.17 (t, 2H, CH_2_ CH_2_), 4.41 (t, 2H, O CH_2_), 3.4 (s, 3H, CH_3_); ^13^C-NMR (DMSO-*d*_6_) δ ppm: 156.09 (N=C–CH3) pyridinium carbons (145.94, 134.67, 128.02, 119.01), 155.77 (C=C–O), naphthalene (129.94, 129.77, 129.77, 129.77, 128.38, 126.58, 124.27, 125.1, 109.18, 107.62), spacer 68.48 (O–CH_2_), 60.4 (O–CH_2_–CH_2_), 31.83 (CH_3_); Anal. Calcd for C18H18BrNO + (343.06): C, 62.80; H, 5.27 Br, 23.21; N, 4.07,O, 4.65; Found: 62.82; H, 5.13; N, 4.08.

#### 3-methyl-1-(2-(naphthalen-2-yloxy) ethyl) pyridin-1-ium Bromide (IL-3MPyBr)

IR (KBr, ʋ cm^−1^ 1215 (C–O), 1389–1457 (C–N), 1598 (C=C), 2940 (CH Aliphatic), 3050 (CH Aromatic); ^1^ H NMR (DMSO-d^6^) δ ppm: pyridinium protons 8.09–9.74 (m, 4H), naphthalene protons 7.12–7.79 (m, 7H) , spacer protons 4.42 (t, 2H, O CH_2_), 5.11 (t, 2H, CH_2_ CH_2_), 3.41 (s, 3H, CH_3_); ^13^C-NMR (DMSO-*d*_6_) δ ppm: 155.77 (N=C–CH3) pyridinium carbons (145.94, 135.07, 128.02, 119.1), 156.09 (C=C–O), naphthalene (129.99, 129.77, 129.75, 129.26, 129.13, 126.93, 124.26, 123.09, 109.24, 107.81), spacer 68.48 (O–CH_2_), 60.4 (O–CH_2_–CH_2_), 31.83 (CH_3_); Anal. Calcd for C18H18BrNO + (343.06): C, 62.80; H, 5.27 Br,23.21; N, 4.07,O, 4.65; Found: 62.70; H, 5.15; N, 4.10.

#### 4-methyl-1-(2-(naphthalen-2-yloxy)ethyl)pyridin-1-ium Bromide (IL-4MPyBr)

IR (KBr,ʋ cm^−1^ 1214 (C–O), 1389–1457 (C–N), 1598 (C=C), 2943 (CH Aliphatic), 3049 (CH Aromatic); ^1^ H NMR (DMSO-d^6^) δ ppm: 3.8 (s, 3H, CH_3_), spacer protons 4.63 (t, 2H, O CH_2_), 5.12 (t, 2H, CH_2_ CH_2_), naphthalene protons 7.13–7.79 (m, 7H) , pyridinium protons 7.81–9.9 (m, 4H); Anal. Calcd for C_18_H_18_BrNO^+^ (343.06): C, 62.80; H, 5.27 Br, 23.21; N, 4.07, O, 4.65; Found: 62.67; H, 5.30; N, 4.09.

### Thermal stability

**Figure 3:**
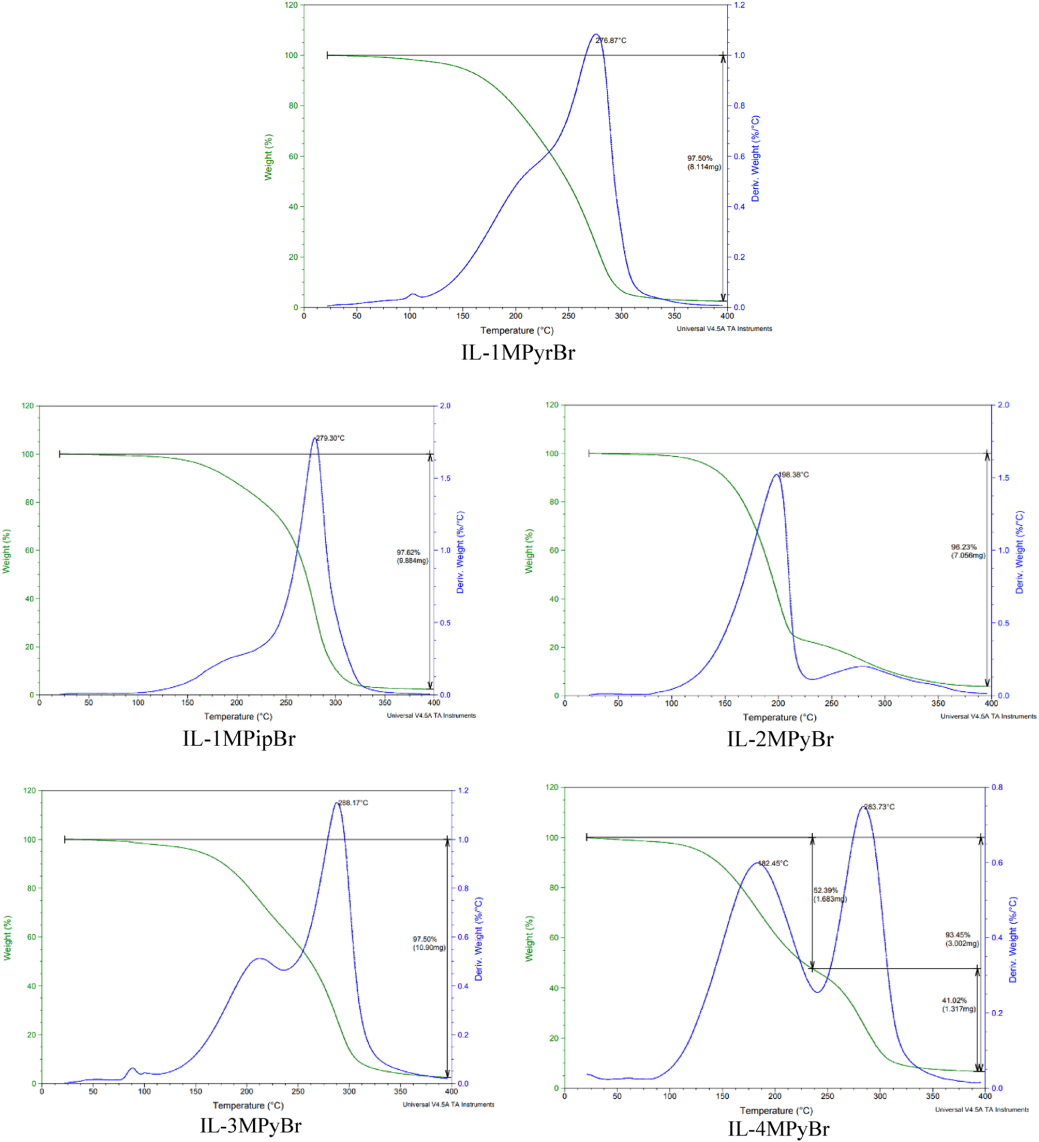
Thermal stability of the synthesized ILs.

Thermal stability was confirmed via studying (TGA) and DTG composite pictures of the synthesized compounds. ILs were shown to have high onset temperatures, which may indicate that they are resistant to thermal degradation. Figure 3 illustrated that IL-1MPyrBr, IL-1MPipBr, IL-2MPyBr, IL-3MPyBr, IL-4MPyBr are thermally stable and firstly decomposed between 198 and 288 °C and finally decomposed up to 400 °C; the endothermal phenomenon was noticed till 100% sample weight loss^28,39,40^. The position of CH_3_ group on the pyridinium ring influence the thermal stability; in case of IL-3MPyBr (meta position), the compound demonstrated greater thermal stability more than IL-4MPyBr and IL-2MPyBr (para and ortho position in order) related to the mesomeric resonance effect, which resist C-bond leakage.

### Anti-corrosion characteristics of the synthesized ILs

#### Electrochemical methods

**Figure 4:**
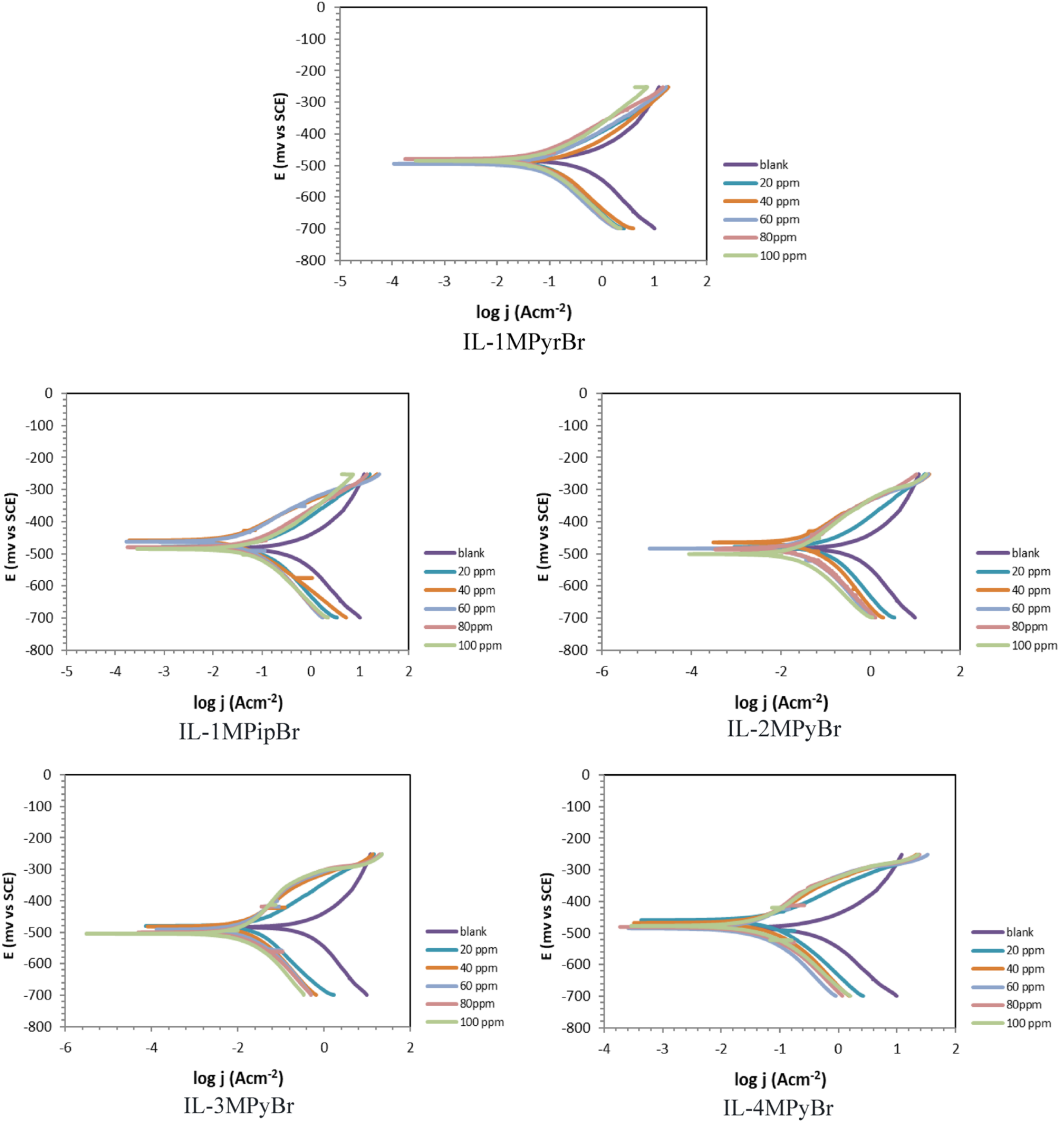
Curves of Potentiodynamic polarization for carbon steel immersed in 1M HCl solution at 298 K.

In the case of IL-1MPyrBr, IL-1MPipBr, IL-2MPyBr, IL-3MPyBr, IL-4MPyBr, the polarization curves are depicted in Fig. 4. Tafel is the format of the plot documents the polarization characteristics, which include the corrosion potential (Ecorr), Tafel slopes (βa and βc), and corrosion current density (jcorr). The following formula is used to determine the protection efficiency (Pj%)^41^.

In a blank acid solution, the corrosion current density jcorr (0) represents the initial corrosion rate. Table 1 provides the following insights:

The jcorr values are notably reduced to very low levels by the presence of ILs.
Compared to the blank solution, there has been less than a 35 mV shift in Ecorr. This suggests that the synthesized ILs exhibit heterogeneous characteristics^41–43^.
3. The percentage of Pj varies from 76.43 to 97.56% at 298 K.
Higher Pj% values are observed at low ILs concentrations. Additional concentrations of ILs do not significantly affect the ILs efficacy. The propensity of the newly created ILs to adsorb on carbon steel specimens is the key factor behind the reduced corrosion rate in a 1M HCl electrolyte.
These ILs are able to inhibit anodic and cathodic processes at the same time. Heteroatoms and p-electrons are the main adsorption centres on the surface of carbon steel^44,45^.

**Table 1:** Effect of ILs concentrations on the polarization variables and inhibition efficiency on carbon steel immersed 1 M HCl solution.

Cpd	Conc. Ppm	Ecorr mV versus SCE		jcorr (μAcm^−2^)		βa (mV dec^−1^)		Βc (mV dec^−1^)		Pj%
	Blank	− 483.30	(± 0.80)	451.60	(± 0.92)	113.30	(± 0.43)	− 171.60	(± 0.85)	
IL-1MPyrBr	20	− 484.00	(± 0.37)	95.53	(± 0.66)	89.90	(± 0.57)	− 165.00	(± 0.80)	78.84
	40	− 494.40	(± 0.64)	85.10	(± 0.50)	69.10	(± 0.45)	− 134.30	(± 0.37)	81.15
	60	− 479.50	(± 0.21)	78.60	(± 0.61)	99.30	(± 0.37)	− 163.00	(± 0.86)	82.59
	80	− 482.30	(± 0.92)	70.90	(± 0.45)	94.60	(± 0.52)	− 152.30	(± 0.82)	84.30
	100	− 494.00	(± 0.43)	68.00	(± 1.01)	93.40	(± 0.92)	− 149.90	(± 0.45)	84.90
IL-1MPipBr	20	− 478.80	(± 0.42)	106.40	(± 0.45)	102.30	(± 0.49)	− 159.40	(± 0.92)	76.43
	40	− 461.80	(± 0.21)	25.14	(± 0.37)	84.60	(± 1.68)	− 84.10	(± 0.38)	94.43
	60	− 458.00	(± 0.29)	20.10	(± 0.31)	75.20	(± 0.35)	− 71.40	(± 0.49)	95.54
	80	− 472.50	(± 0.21)	19.35	(± 0.41)	101.60	(± 0.94)	− 103.30	(± 0.42)	95.71
	100	− 460.40	(± 0.24)	16.63	(± 0.27)	84.60	(± 0.71)	− 78.80	(± 0.49)	96.31
IL-2MPyBr	20	− 484.40	(± 0.29)	87.43	(± 0.65)	95.00	(± 0.35)	− 149.70	(± 0.43)	80.63
	40	− 464.00	(± 0.16)	25.79	(± 0.48)	93.80	(± 0.82)	− 86.10	(± 0.57)	94.28
	60	− 500.50	(± 0.35)	19.85	(± 0.58)	105.40	(± 0.50)	− 101.90	(± 0.35)	95.60
	80	− 483.60	(± 0.21)	18.93	(± 0.30)	94.70	(± 0.86)	− 89.60	(± 0.45)	95.80
	100	− 480.50	(± 0.50)	18.53	(± 0.16)	103.00	(± 0.63)	− 88.90	(± 0.92)	95.89
IL-3MPyBr	20	− 480.10	(± 0.35)	26.36	(± 0.59)	85.80	(± 0.58)	− 137.10	(± 0.49)	94.16
	40	− 481.60	(± 0.45)	15.86	(± 0.36)	118.80	(± 0.58)	− 122.00	(± 1.02)	96.48
	60	− 500.50	(± 0.37)	12.92	(± 0.53)	140.50	(± 0.37)	− 106.40	(± 1.06)	97.13
	80	− 504.70	(± 0.32)	11.66	(± 0.19)	138.20	(± 0.29)	− 105.90	(± 0.35)	97.41
	100	− 491.40	(± 0.37)	11.00	(± 0.49)	111.00	(± 0.29)	− 88.80	(± 0.92)	97.56
IL-4MPyBr	20	− 459.60	(± 0.99)	65.54	(± 0.32)	89.10	(± 0.37)	− 133.00	(± 0.75)	85.48
	40	− 469.10	(± 0.57)	48.43	(± 0.37)	121.50	(± 0.81)	− 119.00	(± 0.26)	89.27
	60	− 476.70	(± 0.69)	40.37	(± 0.29)	146.30	(± 0.43)	− 114.60	(± 0.75)	91.05
	80	− 477.40	(± 0.45)	40.25	(± 0.27)	133.10	(± 0.40)	− 110.60	(± 0.20)	91.08
	100	− 485.00	(± 0.43)	37.74	(± 0.35)	140.30	(± 0.49)	− 130.90	(± 0.35)	91.64

The resulted data showed that IL-1MPipBr and IL-3MPyBr obtain the best adsorption, whereas IL-1MPyrBr and IL-4MPyBr perform the worst. This implies that the adsorption efficacy of the synthesized ILs is directly influenced by the cationic component of their interfacial structure^11^.

**Figure 5:**
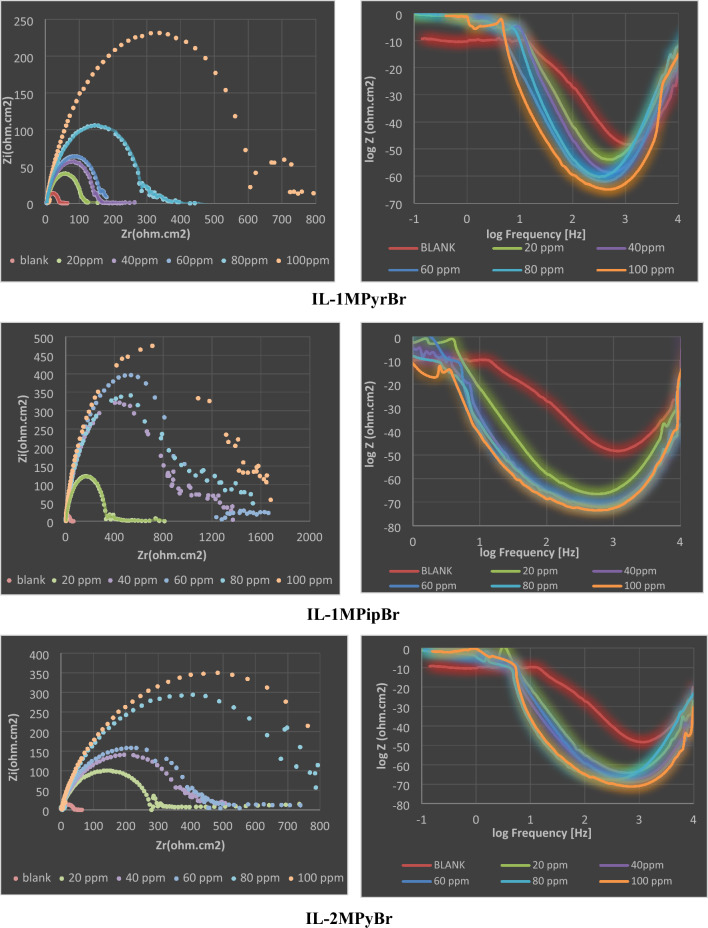

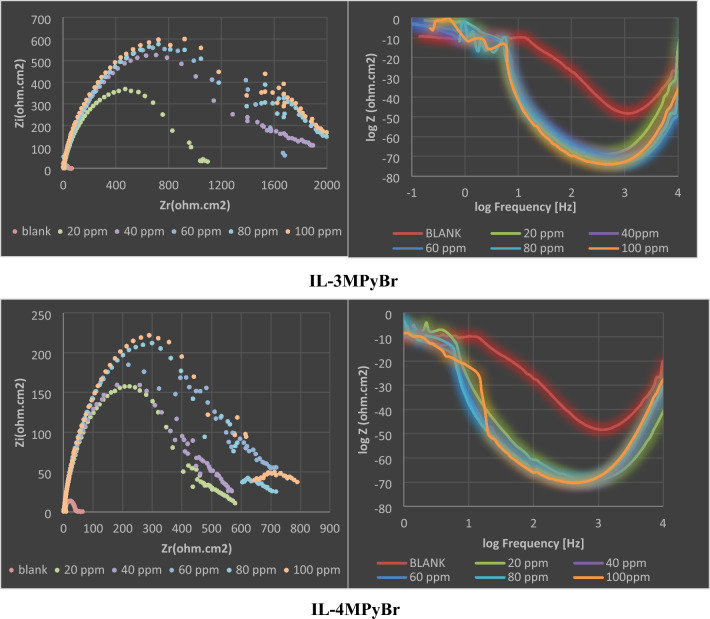
Impedance spectra includes Nyquist, Bode-phase angle for carbon steel immersed in 1 M HCl solution at 298 K for the synthesized ILs.

**Figure 6:**
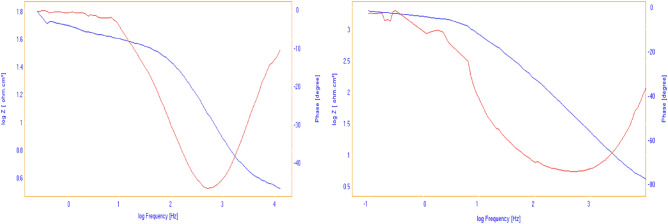
Bode-phase angle & module plots for carbon steel immersed in 1 M HCl solution at 298 K for 100 ppm concentration for blank and 1MPyrBr.

Comprehensive investigation into the effectiveness of newly developed ILs was conducted through Electrochemical Impedance Spectroscopy (EIS) measurements. Figure 5 Illustrates specific Nyquist and Bode-phase angle plots for the five synthesized ILs and Fig. 6 represents bode-phase angle & module plots for carbon steel immersed in 1 M HCl solution at 298 K for 100 ppm concentration for blank and 1MPyrBr. The semicircle indicates that charge transfer process at the electrode/solution interface mainly controlled the corrosion of the metal in the studied medium. Clearly, the diameter of the Nyquist graph increases with increasing ILs concentrations, which suggests slower rate of charge transfer and better protection of the electrode surface probably due to the strengthening of the protective layer. The effect of concentration is also seen in the Bode Phase representation, i.e. the Phase angle is shifted towards higher value with increase in concentration. The shape of the Nyquist and Bode plots are the same in the unprotected and protected systems and indicates same corrosion mechanism.

**Figure 7:**
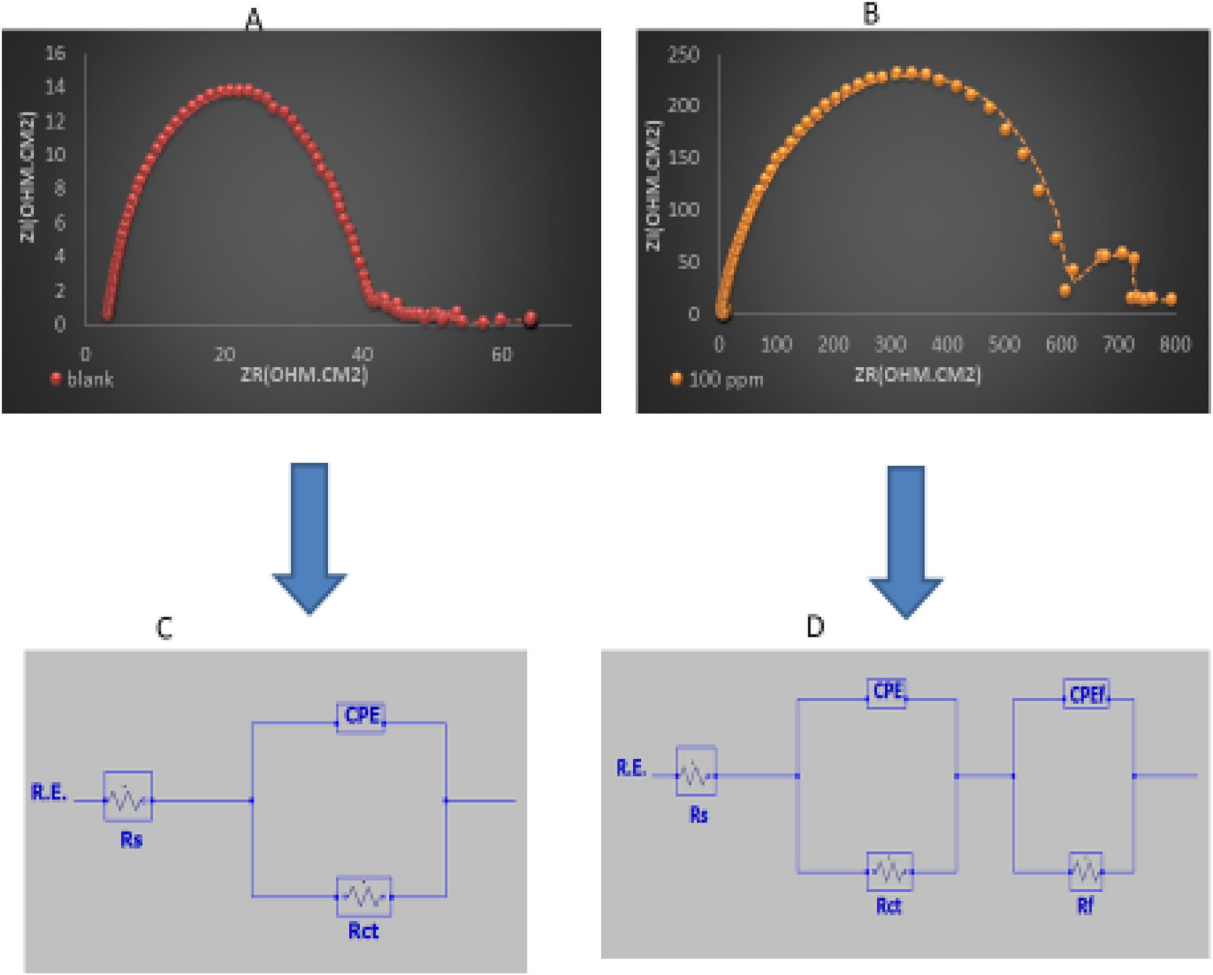
The selected Equivalent Circuits describing EIS data (**A**,**C** for blank and **B**,**D** for 100 ppm concentration of 1MPyrBr).

The equivalent circuit that describe the EIS data is shown in Fig. 7 It consists of *R*s (solution resistance), *Rct* (charge transfer resistance), a CPE (constant phase element) and the film resistance element *Rf*. CPE is used instead of an ideal capacitor due to the heterogeneity of the surface as reflected by the imperfectness of the semicircles^46^.

Refer to all EIS parameters Table 2, the Rs remains consistently low across solutions because of the high conductivity of the studied ILs. By increasing the concentrations of ILs, the magnitude of Rct steadily rises until achieving maximum efficacy. To calculate the protection effectiveness (ηR %), the following equation was employed^47^:

**Table 2:** EIS variables and inhibition efficiency of carbon steel immersed 1 M HCl solution as a function of ILs concentrations.

Cpd	Conc. Ppm	Rs (Ω cm2)		Rct (Ω cm2)		CEPdl (μF cm − 2)		ηR %
	Blank	2.92	(± 0.24)	38.30	(± 0.49)	37.03	(± 0.22)	–
IL-1MPyrBr	20	4.58	(± 0.26)	105.05	(± 0.62)	24.01	(± 0.23)	63.54
	40	3.82	(± 0.08)	147.79	(± 0.39)	21.48	(± 0.26)	74.08
	60	3.80	(± 0.12)	163.45	(± 0.48)	30.79	(± 0.22)	76.56
	80	2.98	(± 0.15)	299.19	(± 0.48)	23.76	(± 0.23)	87.19
	100	1.66	(± 0.22)	688.6	(± 0.34)	16.36	(± 0.17)	94.43
IL-1MPipBr	20	1.54	(± 0.08)	351.22	(± 0.40)	20.24	(± 0.16)	89.09
	40	5.86	(± 0.10)	872.33	(± 0.42)	16.26	(± 0.29)	95.60
	60	1.85	(± 0.21)	985.25	(± 0.32)	20.33	(± 0.26)	96.11
	80	8.65	(± 0.09)	1107.7	(± 1.02)	16.12	(± 0.31)	96.54
	100	2.68	(± 0.22)	1155.7	(± 0.64)	17.33	(± 0.23)	96.68
IL-2MPyBr	20	1.51	(± 0.43)	287.77	(± 0.38)	22.01	(± 0.33)	86.69
	40	0.53	(± 0.12)	406.2	(± 0.26)	24.71	(± 0.16)	90.57
	60	1.22	(± 0.31)	445.03	(± 0.36)	17.88	(± 0.24)	91.39
	80	5.10	(± 0.19)	782.59	(± 0.89)	22.81	(± 0.33)	95.10
	100	6.97	(± 0.21)	996.51	(± 0.22)	15.97	(± 0.27)	96.15
IL-3MPyBr	20	3.88	(± 0.08)	1010.8	(± 0.45)	17.66	(± 0.25)	96.21
	40	15.81	(± 0.20)	1686.2	(± 0.75)	9.43	(± 0.29)	97.72
	60	14.53	(± 0.22)	1544.9	(± 0.53)	11.55	(± 0.33)	97.52
	80	16.6710	(± 0.16)	1662	(± 0.84)	10.74	(± 0.24)	97.69
	100	17.76	(± 0.60)	1669.5	(± 0.27)	12.00	(± 0.54)	97.70
IL-4MPyBr	20	2.17	(± 0.12)	437.31	(± 0.37)	25.76	(± 0.27)	91.24
	40	3.45	(± 0.41)	442.47	(± 0.67)	40.35	(± 0.20)	91.34
	60	3.90	(± 0.65)	566.89	(± 0.33)	28.07	(± 0.19)	93.24
	80	0.33	(± 0.21)	569.01	(± 0.31)	27.97	(± 0.2)	93.26
	100	3.25	(± 0.12)	628.68	(± 0.23)	28.40	(± 0.25)	93.90

1 $$\mathrm{\eta R\%}=\frac{{\text{Rct}}-{\text{Rcto}}}{{\text{Rct}}}*100$$

(Rct0 = charge transfer resistance in the blank solution) In general, raising the concentrations of the synthesized ILs results in an enhancement of the ηR% value, particularly up to effective concentrations as indicated in Table 2. Notably, when IL-3MPyBr was at 100 ppm, the maximum ηR % (97.70%) was achieved.

It is worth mentioning that the reported percentages of corrosion inhibition derived from both polarization and EIS studies exhibit a consistent pattern. The remarkable ability of the synthesized compounds to adsorb to the metal surface plays a major role in diminishing the corrosion rate of carbon steel specimens immersed in a 1M HCl solution^48^. The versatile action of ILs encompasses the suppression of both cathodic and anodic processes simultaneously. The adsorption sites on the steel surface are primarily attributed to oxygen (O), nitrogen (N) atoms, and π-electrons. Data analysis shows that among the studied ionic liquids, IL-3MPyBr emerges as the most efficient, whereas IL-4MPyBr demonstrates the lowest performance. This observation further confirms that the cationic component of the ILs significantly influences its adsorption capacity^49^.

#### Weight loss method

**Figure 8:**
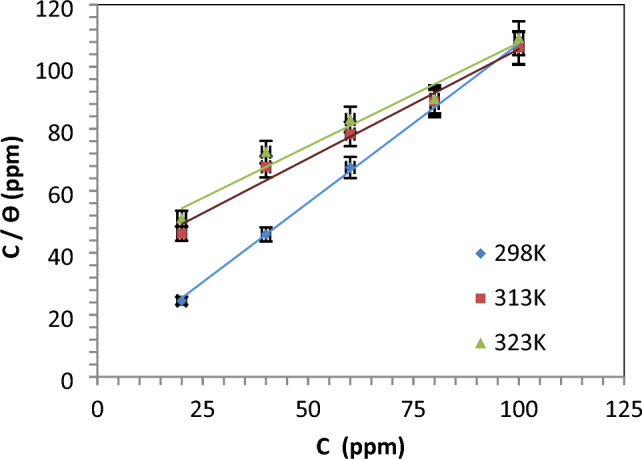
Langmuir isotherm of the studied ILs at different temperatures.

Concentrations of IL-3MPyBr and inhibitory effect: Weight loss-time curves of the carbon steel samples before and after the addition of the synthesized IL are shown in Fig. 8. The curves demonstrate that when the ILs concentrations in the acidic solution increase, the weight losses values (mg.cm^−2^) of the carbon steel decrease, which enhances the inhibitor efficacy to impede corrosion. It is indicated that the formation of complex between ILs-donating species and carbon steel surface resulting the dissolution hindrance. The corrosion rate can be estimated using the inserted equation ^50^.

**Figure 9:**
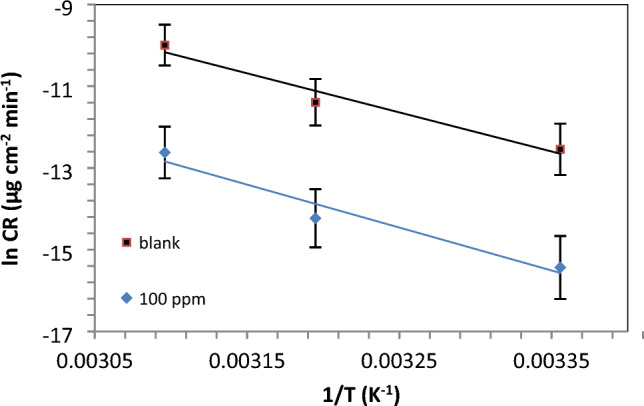
Arrhenius plot for the immersed carbon steel in 1 M HCl without and with IL-3MPyBr (100 ppm).

Figures 8 and 9 shows weight loss-time curves for the carbon steel samples both before and after introducing the synthesized IL. The curves illustrate that as the concentrations of IL-3MPyBr in the acidic solution increase, the weight loss values (in mg.cm^−2^) of the carbon steel decrease. This indicates an improved inhibitory effect on corrosion. The observed trend suggests the formation of complexes between IL-donating species and the surface of the carbon steel, leading to hindered dissolution. The inhibition efficiency (Pw%) of the applied IL-3MPyBr on the carbon steel surface at various studied concentrations was determined in relation to the calculated corrosion rate, as depicted in the inserted equations:

2 $$ W_{corr} = \frac{{W_{1} - W_{2} }}{S \times t} $$

The equation involves the mass losses, denoted as W1 and W2, which pertain to samples of C-steel before and after immersion in acid. S denotes the area of C-steel, and t signifies the immersion time.

3 $$ P_{w} \% = \frac{{W_{corr}^{0} - W_{corr} }}{{W_{corr}^{0} }} \times 100 $$

The corrosion rates of C-steel in the acid solution are represented as W0corr and Wcorr, corresponding to situations without and with the presence of IL, respectively. The collected mass loss data is summarized in Table 3.

**Table 3:** Corrosion rate and inhibition efficiency of carbon steel immersed 1 M HCl solution containing ionic liquid IL-3MPyBr as a function of temperatures.

Cpd	C. ppm	Temperature, K								
		298			313			323		
		*W*_corr_ µg. cm^−2^ min^−1^	θ	*P*_w_ %	*W*_corr_ µg cm^−2^ min^−1^	θ	*P*_w_ %	*W*_corr_ µg cm^−2^ min^−1^	θ	*P*_w_ %
Blank	0	356.29	–	–	1128.27	–	–	3906.04	–	–
IL-3MPyBr	20	65.98	0.8148	81.48	640.01	0.4327	43.27	2375.29	0.3919	39.19
	40	46.18	0.8703	87.03	461.86	0.5906	59.06	1748.48	0.5523	55.23
	60	39.59	0.888	88.88	263.92	0.7661	76.61	1082.08	0.7229	72.29
	80	32.99	0.9074	90.74	112.16	0.9006	90.06	415.67	0.8935	89.35
	100	19.79	0.9444	94.44	65.98	0.9415	94.15	329.90	0.9155	91.55

The influence of temperatures on the inhibitory effect: The corrosion rate of the synthesized ILs, exemplified by IL-3MPyBr, was investigated at various temperature levels to assess the stability of the protective film formed. The impact of temperatures (298, 313, and 323 K) on the weight losses, and consequently on the corrosion rate of carbon steel, both in the absence and presence of IL-3MPyBr at the same concentration of acidic medium (1 M HCl). Temperature plays a significant role in influencing the rate of metal corrosion. In an acidic solution, due to the reduction in hydrogen evolution over potential (hydrogen depolarization), the corrosion rate exponentially increases with temperature^47^. The results indicate that the corrosion rate rises with an increase in temperature, leading to a gradual loss of efficacy in the corrosion inhibition of the carbon steel surface. The corresponding data have been calculated and are presented in Table 3.

Adsorption process investigation: Utilizing data obtained from weight loss tests, we investigated the optimal adsorption isotherm to characterize the adsorption process of the IL-3MPyBr on the surface of steel in 1M HCl. The Gibbs free energy (G°_ads_) associated with the adsorption process and K_ads_ (equilibrium constant of the adsorption/desorption process) are represented in the given equation:

4 $$\frac{{C}_{inh}}{\uptheta }=\frac{1}{{{\text{K}}}_{{\text{ads}}}}+{C}_{inh}$$

5 $$ \Delta G^\circ_{ads} = - {\text{RT}}\;\ln \left( {10^{6} K_{ads} } \right) $$

In the provided equation, where R represents the universal gas constant, and T is the temperature measured in Kelvin. Figure and Table record the Langmuir isotherm and isotherm parameters for the producing ionic IL-3MPyBr.

In this case, the linear regression coefficient (R^2^) parameters approaching one indicate the availability of the isotherm. The relatively small values of K_ads_, suggest that the molecules bonded to the steel surface are predominantly undergoing physical adsorption.

The reported ∆G°^ads^ in Table 4 is − 37.9076 kJ mol^−1^, and this negative value for IL-3MPyBr signifies the spontaneous adsorption on the carbon steel surface ^50^. The range of − 20 to − 40 kJ mol^−1^ suggests an association of both physisorption and chemisorption for the IL on the carbon steel surface.

**Table 4:** Langmuir isotherm parameters of IL-3MPyBr at 298 K.

Cpd	R^2^	K_ads_ (ppm^−1^)	ΔG°_ads_ kJ mol^−1^
IL-3MPyBr	0.99	202.191 × 10^−3^	− 37.9076

The activation energy (E_a_) for the corrosion process can be determined using the following formula from an Arrhenius-type plot:

$$ W_{{{\text{corr}}}} = k\;{\text{exp }}\left( { - E_{{\text{a}}} /RT} \right) $$

**Figure 10:**
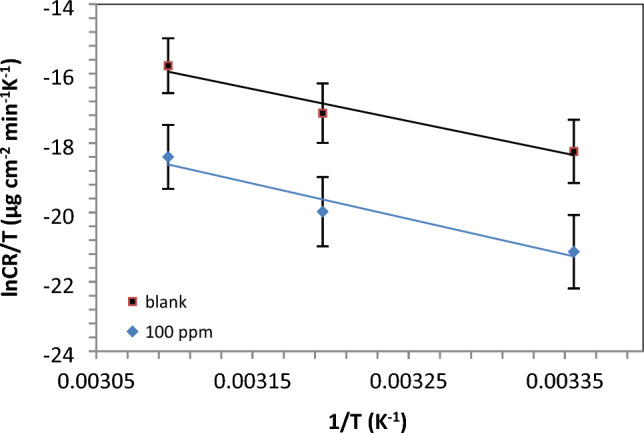
Transition state plot for the immersed carbon steel in 1 M HCl without and with IL-3MPyBr (100 ppm).

In the given equation, W_corr_ represents the corrosion rate, k is the Arrhenius pre-exponential factor, and R and T denote the universal gas constant and absolute temperature, respectively. The calculated values of activation energy (E_a_) are positive, indicating that the corrosion process is endothermic in nature, aligning with previous studies. By comparing to the blank, the activation energy; E_a_ values; Table 5 and Fig. 10 for the systems inhibited by IL-3MPyBr are smaller, providing support for the chemical adsorption mechanism with the prepared IL in 1M HCl solution.

**Table 5:** Carbon steel activation parameters in 1M HCl solution before and after using 100 ppm IL-3MPyBr.

Cpd	C (ppm)	Thermodynamic and kinetic parameters		
		E_a_, kJ.mol^−1^	∆H*, kJ.mol^−1^	∆S*, kJ.mol^−1^.K^−1^
Blank	0	79.3821	12.70696	− 9.25441
IL-3MPyBr	100	60.77119	10.01	− 12.95

The kinetic activation parameters have been assessed using Fig. 9 and Eq. 6.

6 $${W}_{corr}=\frac{RT}{{N}_{A}h}{\text{exp}}\left(\frac{{\Delta S}^{*}}{R}\right){\text{exp}}\left(\frac{{\Delta H}^{*}}{RT}\right)$$

In the provided equation, NA represents Avogadro’s number, h is Planck’s constant, ΔS* is the entropy of activation and ΔH* is the enthalpy of activation. Notably, the positive values of ΔH* signify an endothermic activation process. It's noteworthy that the calculated E_a_ values are slightly larger than the apparent activation enthalpy (ΔH*). The ΔS* values for the IL-3MPyBr system are more negative compared to the value obtained for the uninhibited acid system, indicating that the IL plays an effective role against the corrosion process. This suggests a reduction in disturbance as low carbon steel dissolves in systems with inhibitors.

### Theoretical study

The computed Tables 6 and 7 and the experimental results were agreed entirely. As shown in Table 6 the values of ΔE_back-donation_ for the synthesized ammonium based ILs recorded (− 0.669, − 0.656, − 0.828, − 0.664& 0.827). One of the most important indicators of the corrosion inhibition process is the dipole moment^51^. Increasing the dipole moment leads to an improvement in strain energy and an increase in molecule adsorption on the metal surface. Therefore, increasing the dipole moment increases the effectiveness of corrosion inhibition^52^. As mentioned in Table 7 IL-3MPyBr has a larger dipole moment value (17.1497 Debye) than IL–IL-1MPipBr, IL-1MPyrBr, IL-2MPyBr and IL-4MPyBr which recorded values as 17.1383, 15.9486, 15.8485 and 14.6848 Debye in order. Showing that the IL-3MPyBr's capacity to adhere to the surface of carbon steel and enhance inhibition is stronger^37,53^. Furthermore, the selection of inhibitory molecules to protect carbon steel's surface from corrosive environments is influenced by the molecular surface of the material^46^.

**Table 6:** The calculated quantum parameters for the synthesized cyclic ammonium based ILs.

	E_HOMO_ (eV)	E_LUMO_ (eV)	ΔE (eV)	Dipole moment, µ ( Debye)	Electron affinity, A (eV)	Ionization potential, I (eV)	Electro-negativity (eV mol^−1^)	Hardness, ɳ (eV mol^−1^)	Softness (eV^−1^)	ΔE _back-donation_
IL-1MPyrBr	− 10.768	− 5.414	5.354	15.9486	5.414	10.768	8.091	2.677	0.3735525	− 0.669
IL-1MPipBr	− 10.653	− 5.403	5.25	17.1383	5.403	10.653	8.028	2.625	0.3809524	− 0.656
IL-2MPyBr	− 10.744	− 4.12	6.624	15.8485	4.12	10.744	7.432	3.312	0.3019324	− 0.828
IL-3MPyBr	− 10.671	− 5.36	5.311	17.1467	5.36	10.671	8.0155	2.6555	0.3765769	− 0.664
IL-4MPyBr	− 10.714	− 4.094	6.62	14.6848	4.094	10.714	7.404	3.31	0.3021148	− 0.827

**Table 7:**
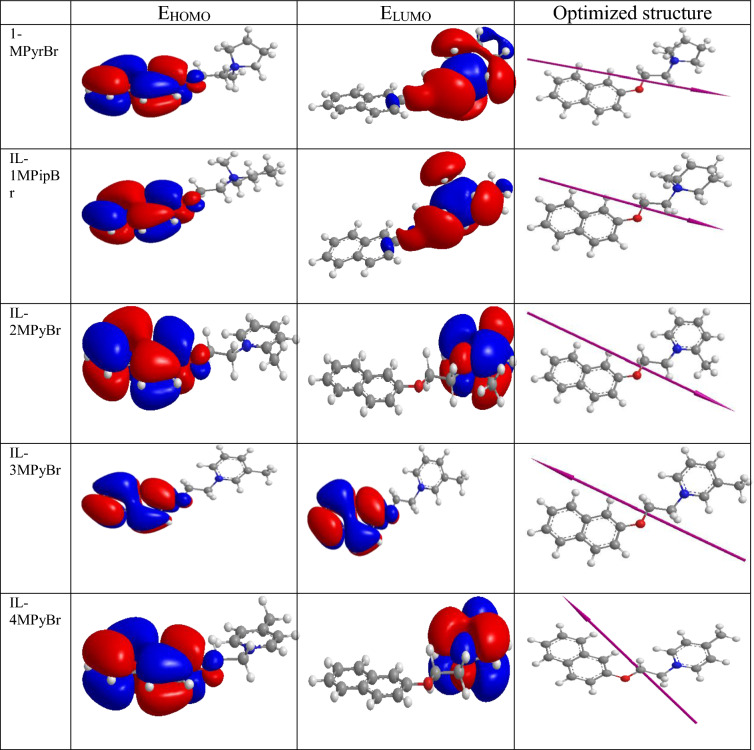
E_HOMO_, E_LUMO_ and the optimized molecular structures for the synthesized cyclic ammonium based ILs.

### Effect of Structure on the inhibitory performance

The five synthesized ILs possess anionic part (Br^−^) and five different cationic hetero rings (N-methylpyrrolidinium, N-methylpipredinium and three pyridinum derivatives). The main reason for the inhibition efficiency differences is the structure of the catationic part ^50^. IL-1MPyrBr and IL-1MPipBr in which the cations are saturated, whereas IL-2PyBr, IL-3MPyBr and IL-4MPyBr having aromatic pyridinium cations. The planarity of the pyridinium ring makes IL-2PyBr, IL-3MPyBr and IL-4MPyBr spread well on the metal surface since, they record more corrosion inhibition than IL-1MPyrBr and IL-1MPipBr. Moreover, the six membered ring is less strained ring than the five membered ones, so the binding force between the metal and IL-2PyBr, IL-3MPyBr & IL-4MPyBr is greater than that between the metal surface and IL-1MPyrBr &IL-1MPipBr. Also, for IL-2PyBr, IL-3MPyBr & IL-4MPyBr, the presence of Me group in meta-position lets it free and less affected by the electron with drawing pyridinium ring than in para- and ortho-positions, so IL-3MPyBr records the best corrosion efficiency using the lower concentration (20 ppm)^54^.

### Inhibition performance after acid-cleaning operations using IL-3MPyBr

As a semi-pilot bench scale for the industrial application; the cleaning process^47^ could be performed by involving the immersion of materials in a hydrochloric acid solution, with a carefully chosen concentration (1M). IL-3MPyBr are introduced to mitigate the potential corrosive effects of the acid. The influence of critical parameters, including acid concentration (1M), temperature (40 °C), and inhibitor dosage (100 ppm) for definite time (72 h), is systematically investigated to optimize the cleaning process. Table 8 showed that the inhibition performance is 97.41%.

**Table 8:** Inhibition performance after acid-cleaning operations.

	Wt, before, g	Wt After 72 h, g	Wt loss, g	*P*_w_ %
Blank	5.8539	5.4213	0.4326	–
IL-3MPyBr	5.8366	5.8254	0.0112	97.41

### Mechanism of inhibition

**Figure 11:**
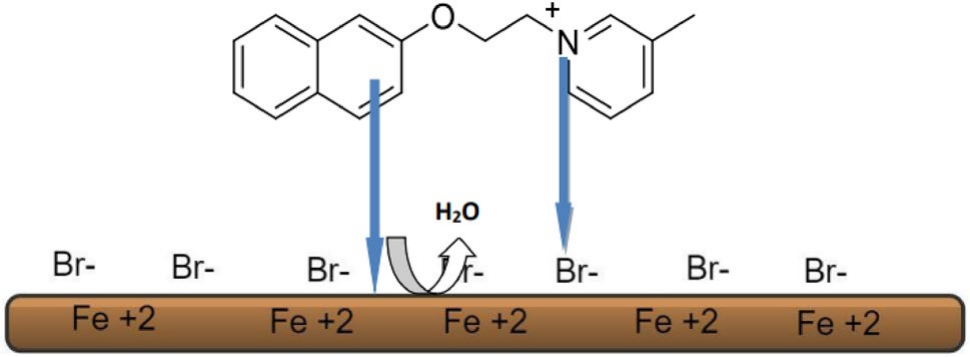
Mechanism of inhibition.

The mechanism by which ILs inhibit corrosion can be elucidated in Fig. 11 as the attachment of ILs' donating components onto the anodic sites of carbon steel, forming a layer that adheres to the surface and enhances protection against corrosion. ILs possess the capability to displace water molecules adsorbed on the surface of C-steel, functioning similarly to commercial inhibitors. Initially, a physisorption process occurs as the positively charged cations on metal surfaces interact electrostatically with the anions of the ILs. Additionally, physisorption may happen through the interaction between anionic species on metal surfaces and the cationic portions of the ILs under study. It is hypothesized that at cathodic sites on the metal surface, the larger cations of ILs can readily replace the adsorbed water molecules. This results in enhanced protection against corrosion, facilitated by the presence of hetero-organic moieties that offer additional protective potential and long chains that increase coverage per molecule.

This interaction involves coordination bonds as IL molecules adhere to form a restrictive layer at the interface between metal and solution. The presence of N=`C–N moieties provides active sites for adsorption on the metal surface, and the adsorption process may be intensified by the presence of multiple bonds. Moreover, the proposed chemical adsorption process involves interactions where the π-electrons on the nitrogen atom of the imidazolium ring and the π-electrons of the aromatic moieties interact with the vacant d-orbital of the carbon steel, thus preventing its dissolution in the aggressive medium. It has also been suggested that adsorption may occur through the orientation of the cation species of the ILs parallel to the metal surface. The delocalization of the nitrogen atom of the cation within the ring creates a mild positive charge on the entire IL cation. Consequently, the IL cation seeks to accept electrons from the carbon steel surface. This process can alter the polarity of the carbon steel surface by forming a multilayer of the adsorbed species on the surface. Additionally, the aromatic moieties of the IL donate π-electrons to the vacant d-orbitals on the surface of the carbon steel, resulting in the formation of donor–acceptor complexes. These preceding processes generate an inter-electronic repulsion between the IL and the metal surface, facilitating back bonding or retro-donation. The donation of electrons and back donation strengthen one another, effectively blocking the surface of the carbon steel and subsequently preventing corrosion. This creates a stable chemical coating that reduces friction and wear^53^.

## Conclusion

In conclusion, series if cyclic ammonium based ionic liquids (IL-1MPyrBr, IL-1MPipBr, IL-2PyBr, IL-3MPyBr, and IL-4MPyBr) were synthesized and well confirmed via variety spectroscopic techniques to prevent corrosion of C-steel in 1 M HCl and understanding the compounds' structure–property relationship. The anti-corrosion properties of the synthesized ILs were being assessed by weight loss, chemical and electrochemical parameters (PDP and EIS). According to the current study, all these inhibitors has exceptional ability to prevent the corrosion. At low and high concentration; 20 and 100 ppm, IL-3MPyBr has the maximum corrosion inhibition efficacy of 94.16 and 97.56% respectively. The experimental results were in complete combatable with the theoretical, which showed that, IL-3MPyBr has a larger dipole moment value (17.1497 Debye) showing its capacity to adhere to the surface of carbon steel and enhance inhibition. These observed results will motivate a number of ongoing researchers to put in endless effort to uncover the anti-corrosion properties of new ILs in the acid additives markets.

## Data Availability

The data that support the findings in the present study are available from the corresponding author upon request.
